# Unique Substrate Recognition and Sodium–Substrate Binding Stoichiometry in a Bacterial Serotonin Transporter, TuriSERT

**DOI:** 10.3390/ijms242317112

**Published:** 2023-12-04

**Authors:** Mu Li, Xintong Zhang, Sixiang Chen, Hanhe Liu, Yuan-Wei Zhang

**Affiliations:** School of Life Sciences, Guangzhou University, Guangzhou 510006, China; limu2021@gzhu.edu.cn (M.L.); 2112214037@e.gzhu.edu.cn (X.Z.); 2112114040@e.gzhu.edu.cn (S.C.); jokerliuhh@e.gzhu.edu.cn (H.L.)

**Keywords:** monoamine transporter, neurotransmitter transporter, serotonin transporter, bacterial transporter, substrate and ion binding, stoichiometry, antidepressants, 5-hydroxytryptamine

## Abstract

All resolved high-resolution structures of the transporters in the neurotransmitter sodium symporter (NSS) family revealed that the NSS members share common structural and mechanistic features for substrate and ion binding and transport. However, a recently reported bacterial orthologue of the human serotonin transporter (hSERT), TuriSERT, possesses a structural characteristic specific for amino acid substrate binding but does transport a biogenic amine. The unique structural feature of TuriSERT requires a novel configuration for coordinating its substrate and ions. In the present study, we characterized TuriSERT expressed in *Escherichia coli* cells with a fluorescent substrate by biochemical, structural, and pharmacological approaches. Substrate transport by TuriSERT requires Na^+^ but not Cl^−^. Replacement of Asp262 by asparagine renders TuriSERT Cl^−^-dependent. Substitutions of the corresponding Na1 residues did not alter Na^+^ dependence on substrate transport, whereas the mutation of a Na2 site residue led to a loss of transport activity, suggesting that Na^+^ binds only to the Na2 site in TuriSERT. In addition, substitutions of several residues essential for recognizing 5-hydroxytryptamine (5-HT) in hSERT had little effect on 5-HT displacement potency in transport assay for TuriSERT. In contrast, mutations of the residues that are proposed to coordinate with 5-HT in our docking model dramatically reduced 5-HT displacement. Furthermore, our results indicated that all tested antidepressants showed a weak inhibitory effect on TuriSERT. The present study demonstrated the existence of a unique substrate binding site and 1:1 stoichiometry of sodium–substrate binding in TuriSERT, a novel structural finding for the NSS transporters.

## 1. Introduction

The neurotransmitter sodium symporter (NSS) family comprises a group of prokaryotic and eukaryotic transporters that use pre-existing transmembrane Na^+^ gradient as a driving force to power the downhill ion movement and the uphill movement of their respective substrates [1,2]. Eukaryotic members of the NSS family include transporters for neurotransmitters (biogenic amines and amino acids), which are responsible for the re-uptake of neurotransmitters such as serotonin, dopamine, norepinephrine, γ-aminobutyric acid, and glycine after their secretion into the synapse and play critical roles in neurotransmitter transmission [3,4]. Notably, many eukaryotic members in this family are the therapeutic targets for the treatment of an array of neuropsychiatric diseases, such as depression, anxiety, attention deficit hyperactivity disorder, and schizophrenia [5,6,7,8], and also the targets for drug abuse, such as cocaine and amphetamines [9].

The NSS family transporters share a common structural fold comprising 12 transmembrane domains (TM1-12) and similar transport mechanistic features [10]. The family members are thought to transport their substrates by an alternating access mechanism by which the central substrate binding site is alternately exposed to the extracellular or cytoplasmic medium for substrate binding and release by conformational changes that open and close the substrate permeation pathway [11,12,13]. The high-resolution structures of several NSS transporters have revealed the common binding sites for their substrates and ions. The most striking structural characteristic for substrate binding is the presence of aspartate to neutralize positively charged amines in biogenic amine transporters [14,15,16,17,18], such as Asp98 in mammalian serotonin transporter (SERT) [16,18], whereas the aspartate is substituted by glycine at the corresponding position in amino acid transporters [19,20,21,22,23]. In addition, all NSS structures have been shown to contain two Na^+^ binding sites [10]. Na^+^ binding in the Na2 site is essential for conformational changes [24,25,26]. In contrast, Na^+^ occupation in the Na1 site has been proposed to mediate conformational changes through a network of intermediary interactions in the extracellular pathway [27,28]. Furthermore, while most eukaryotic NSS transporters require both Na^+^ and Cl^−^ to transport substrates [29], their prokaryotic counterparts are Cl^−^-independent [10,21,30,31].

5-Hydroxytryptamine (5-HT), as an important signaling molecule, plays critical roles not only in the central nervous system (CNS) but also in the gastrointestinal tract in which 5-HT is synthesized by enterochromaffin cells and regulated by SERT expressed in the epithelial cells in the lining of the intestines [32,33]. The 5-HT molecule mediates many gut functions, including peristalsis, secretion, vasodilation, and perception of pain or nausea through the activation of various 5-HT receptors [34]. 5-HT also exerts a nonconventional action as a pro-inflammatory signaling molecule that is involved in the pathology of several inflammation-related intestinal disorders, such as inflammatory bowel disease and irritable bowel syndrome [35]. In addition, the 5-HT level in the gastrointestinal tract is regulated by the gut microbiota mainly through their effects on 5-HT biosynthesis [36]. Strikingly, a gut bacterium, *Turicibacter sanguinis*, has been recently identified to express a functional orthologue of the host-expressed SERT, TuriSERT [37]. The finding of TuriSERT has improved our understanding of the versatility of the gut microbiota in the regulation of the gut-derived 5-HT.

TuriSERT shows only 34% sequence identity with human SERT (hSERT). Residues participating in 5-HT and selective 5-HT reuptake inhibitor (SSRI) binding in hSERT are approximately 50% identical to those in its bacterial orthologue. There are differences in several key residues that coordinate Na^+^ (Asn368, replaced by D262 in TuriSERT), Cl^−^ (Asn368, replaced by D262), and 5-HT (Asp98 and Tyr95, replaced by Gly22 and S19, respectively) binding in hSERT. These residue alterations raise the question of how TuriSERT coordinates substrate and ion binding and whether SSRIs effectively inhibit TuriSERT. In the present study, we elucidate ionic requirement, ion and substrate binding, and SSRI inhibition in TuriSERT by using molecular docking, biochemical, and pharmacological approaches. Our results show that TuriSERT functions with a unique configuration for substrate binding and sodium–substrate binding stoichiometry.

## 2. Results

### 2.1. TuriSERT Expressed in E. coli Cells Is Functional for APP^+^ Transport

**Figure 1:**
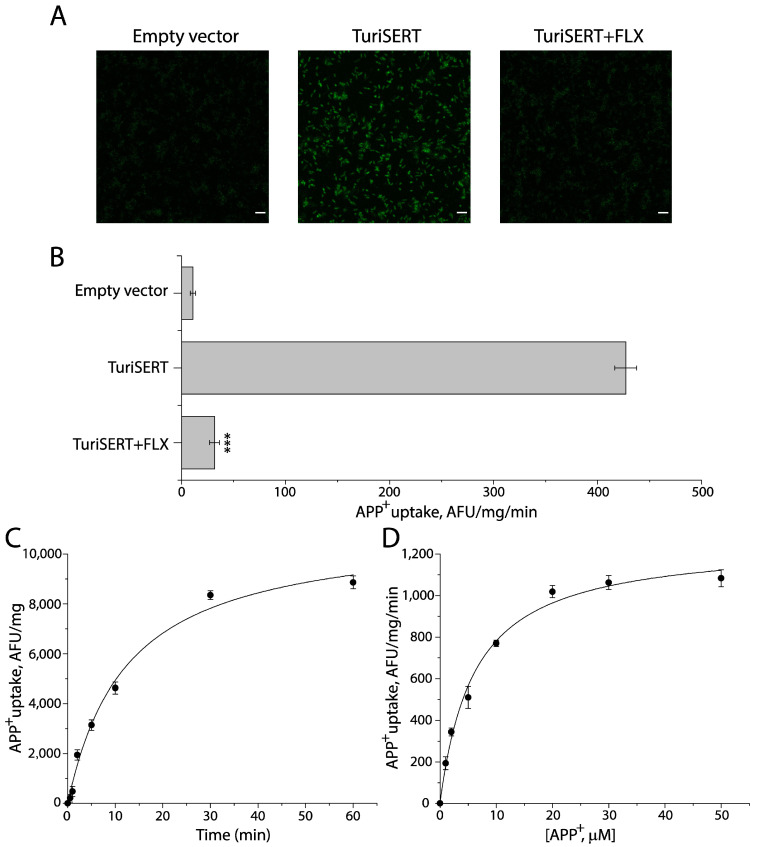
APP^+^ uptake by *E. coli* cells expressing TuriSERT. *E. coli* cells transformed with an empty vector or an expression plasmid for TuriSERT were incubated with APP^+^ in the absence or presence of 1 mM fluoxetine at 37 °C for 5 min. After reactions were terminated by rapid washes, the APP^+^ fluorescence that accumulated in the cells was captured using the Zeiss LSM 900 confocal microscope or determined by a microplate fluorescence reader. (**A**) Representative confocal images of APP^+^ fluorescence accumulated in *E. coli* cells (scale bar: 10 μm). (**B**) Fluoxetine inhibition of APP^+^ transport by TuriSERT expressed as a specific transport activity. The non-specific transport by *E. coli* cells transformed with an empty vector was 10.83 ± 2.58 AFU (arbitrary fluorescence unit)/mg of total proteins from *E. coli* cells/reaction min or 9.65 ± 2.24 AFU/mg/min in the absence or presence of 1 mM fluoxetine, respectively. After subtracting non-specific transport, the specific transport activity of *E. coli* cells expressing TuriSERT was 416.20 ± 10.40 AFU/mg/min. Error bars represent ± SEM (*n* = 3). *** *p* < 0.001 compared to TuriSERT without fluoxetine treatment. (**C**) Time-dependent APP^+^ uptake by TuriSERT. The graph shows a representative experiment. Error bars show ± SD from six measurements. The experiment was repeated twice with similar results. (**D**) Kinetic analysis for APP^+^ uptake by TuriSERT. The graph shows a representative experiment. Error bars show ± SD from six measurements. K_m_ for APP^+^ and V_max_ values shown in the context represent mean ± SEM (*n* = 3). FLX, fluoxetine.

To characterize TuriSERT, we constructed an *Escherichia coli* (*E. coli*) expression system for TuriSERT in which a GST tag and a His tag were fused to the N- and C-termini of TuriSERT, respectively, and then examined its ability to transport a fluorescent substrate of eukaryotic SERT by incubating the *E. coli* cells with 4-[4-(dimethylamino) phenyl]-1-methylpyridinium (APP^+^). As shown in Figure 1A, compared to the control cells transformed with an empty vector, the strong APP^+^ fluorescence accumulated was detected with the cells expressing TuriSERT. The APP^+^ accumulation by TuriSERT was effectively blocked by co-incubation with an SSRI antidepressant, fluoxetine, suggesting TuriSERT expressed in *E. coli* cells was functional for APP^+^ transport. A trace of APP^+^ signal was also seen with the control *E. coli* cells, possibly due to non-specific transport because it was insensitive to fluoxetine treatment. After subtracting non-specific transport determined by fluorescence spectrometry, we obtained specific transport activity of total proteins from *E. coli* cells expressing TuriSERT, which was over 40-fold higher than non-specific transport (Figure 1B).

Figure 1C,D show time course and kinetic analysis for APP^+^ uptake by the cells expressing TuriSERT, respectively. TuriSERT transported APP^+^ into the cells in a time-dependent manner in which the t_1/2_ was estimated to be 13.28 ± 0.36 min. The K_m_ for APP^+^ and V_max_ were 6.12 ± 0.22 μM and 1285 ± 14 AFU/mg/min, respectively.

### 2.2. Ionic Requirement for APP^+^ Uptake by TuriSERT

**Figure 2:**
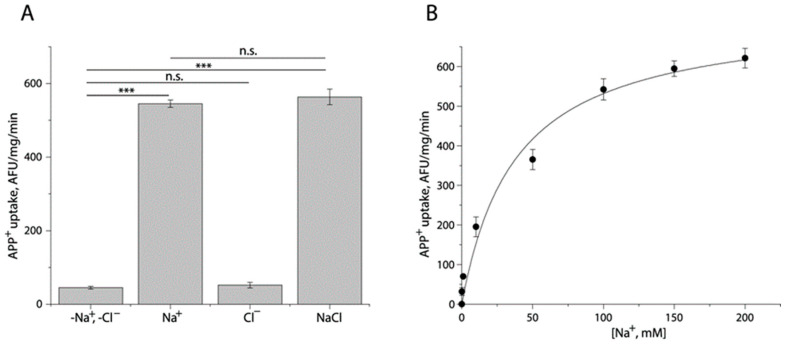
Ionic requirement for APP^+^ uptake by TuriSERT. APP^+^ uptake was assayed by incubating *E. coli* cells expressing TuriSERT with APP^+^ in 20 mM HEPES buffer, pH 7.4, containing 150 mM NMDG gluconate, sodium isethionate, NMDG chloride, or sodium chloride, respectively, as described under Section 4. (**A**) Specific APP^+^ uptake under various ionic conditions. Error bars show ± SEM (*n* = 3). *** *p* < 0.001 compared to samples in the absence of both Na^+^ and Cl^−^ ions. n.s. represents no statistical difference in APP^+^ uptake between samples in the indicated ionic conditions, such as -Na^+^, -Cl^−^ vs. Cl^−^ alone, or Na^+^ alone vs. NaCl. (**B**) Kinetic analysis for Na^+^-dependent APP^+^ uptake by TuriSERT. The graph shows a representative experiment with six measurements. K_m_ value for Na^+^ shown in the context represents ± SEM (*n* = 3).

To examine the ionic requirement for TuriSERT, we employed 20 mM HEPES buffer, pH 7.4, containing 150 mM Na^+^, Cl^−^, or both to measure the ability of TuriSERT to uptake APP^+^ (Figure 2A). Compared to the control in which neither Na^+^ nor Cl^−^ was present, Cl^−^ alone did not drive APP^+^ uptake by TuriSERT. In contrast, TuriSERT in the presence of Na^+^ alone exhibited an ability to transport APP^+^. Noticeably, the addition of Cl^−^ ion to the Na^+^-containing buffer did not show an additive effect on APP^+^ transport, suggesting that APP^+^ transport by TuriSERT is Cl^−^-independent. Figure 2B shows kinetic analysis for Na^+^-driven APP^+^ transport. The K_m_ value for Na^+^ was 33.33 ± 1.33 mM.

### 2.3. Mutation of Asp262 to Asparagine Renders TuriSERT Cl^−^-Dependent

**Figure 3:**
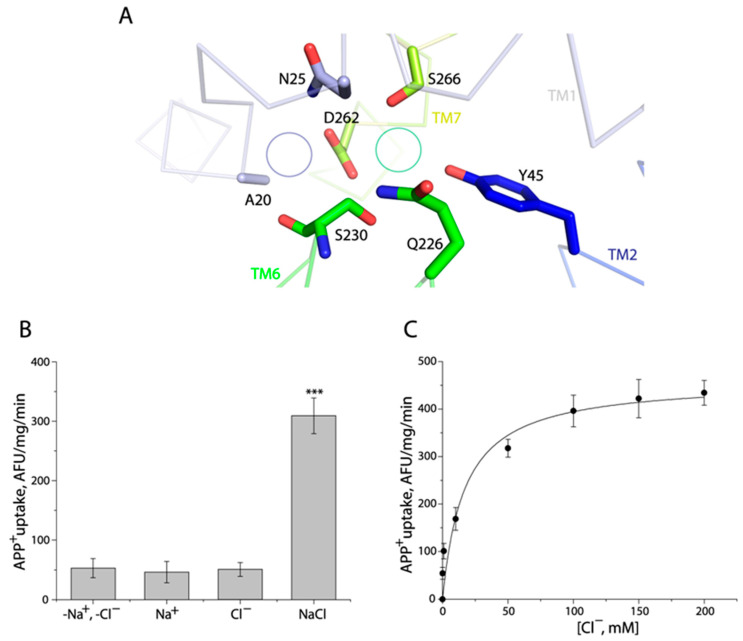
The D262N mutant required both Na^+^ and Cl^−^ for its transport activity. (**A**) A close-up of the region in the TuriSERT model that has been shown to contribute to Na^+^ (Na1) and Cl^−^ binding in the structures of hSERT. The green or purple circles represent the corresponding position of Cl^−^ or Na^+^ (Na1) in hSERT, respectively. (**B**) Ionic requirement for APP^+^ uptake by a D262N mutant. APP^+^ uptake was assayed by incubating *E. coli* cells expressing TuriSERT with APP^+^ in 20 mM HEPES buffer, pH 7.4, containing 150 mM NMDG gluconate, sodium isethionate, NMDG chloride, or sodium chloride, respectively, as described under Section 4. Error bars represent ± SEM (*n* = 3). *** *p* < 0.001 compared to samples in the absence of both Na^+^ and Cl^−^. No statistical difference in APP^+^ uptake was seen between D262N in the indicated ionic conditions, such as -Na^+^, -Cl^−^ vs. Na^+^ alone, or Cl^−^ alone. (**C**) Kinetic analysis for Cl^−^-dependent APP^+^ uptake by a D262N mutant. The graph shows a representative experiment with six measurements. K_m_ value for Cl^−^ shown in the context represents ± SEM (*n* = 3).

The Cl^−^ binding site in human monoamine transporters is completely conserved, comprising five residues, for instance, Tyr121, Gln332, Ser336, Asn368, and Ser372 in hSERT [17]. A comparison of these residues with those in TuriSERT revealed that the corresponding residue of Asn368 is substituted by a negatively charged residue, aspartate, in TuriSERT (Asp262, Figure 3A). Interestingly, this asparagine is also replaced by aspartate in several transporters from bacteria and insects, which are generally Cl^−^-independent [38,39]. We, then, examined the effect of Asp-to-Asn substitution on Cl^−^-independency of APP^+^ transport. As shown in Figure 3B, unlike WT, APP^+^ transport by the mutant D262N required both Na^+^ and Cl^−^ with over 70% of WT activity in the absence of Cl^−^. In addition, the transport activity of D262N was increased in a Cl^−^ concentration-dependent manner with the K_m_ value for Cl^−^ of 16.76 ± 1.53 mM in the presence of 30 mM Na^+^ (Figure 3C). Thus, the insertion of a carboxylate group at this position is proposed to prevent Cl^−^ from stimulating APP^+^ transport, suggesting that TuriSERT does not couple Cl^−^ to the transport substrate.

### 2.4. Mutations of the Na1 Site Do Not Alter Na^+^ Binding Dependence of APP^+^ Transport

The Na1 site in hSERT is formed by side chains of Asn101, Ser336, and Asn386 and main chain carbonyls from Ala96 and Ser336 [16]. Mutating the corresponding residues in LeuT, such as Asn27 or Asn286 with serine and Thr254 with alanine, resulted in profound effects on both transport activity and Na^+^ binding affinity [27]. Among these Na1 residues, there is only one difference from that in TuriSERT, i.e., Asn368 in hSERT is replaced by Asp262 in TuriSERT (Figure 3A). The substitution of Asp262 with asparagine in the bacterial orthologue had a slight effect on its ability to either transport APP^+^ or bind Na^+^ (Table 1). Moreover, we also mutated two other Na1-corresponding residues, Asn25 with serine and Ser230 with alanine in the background of TuriSERT WT (Figure 3A). Strikingly, as shown in Table 1, unlike those in LeuT, these substitutions showed a negligible effect on transport activity, K_m_ value for Na^+^, and 5-HT displacement potency of APP^+^ compared to those in WT. In contrast, the replacement of one Na2 residue, Ser335 with alanine, rendered a mutant transporter nonfunctional, probably due to a loss of the ability to coordinate Na^+^ in the Na2 site. These results suggest that mutations of the Na1 site in TuriSERT did not alter both Na^+^ and 5-HT binding.

### 2.5. Identification of 5-HT Binding Site in TuriSERT

**Figure 4:**
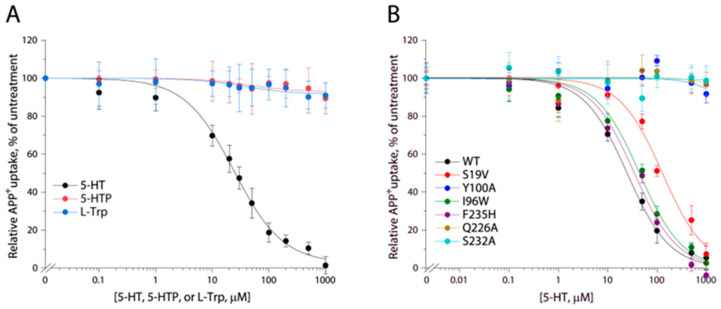
Displacement of APP^+^ in TuriSERT WT and its mutants by 5-HT or its precursors. *E. coli* cells expressing TuriSERT WT or its mutants were incubated with 6 μM APP^+^ and 5-HT, L-tryptophan, or 5-hydroxytryptophan, at the indicated concentrations, and APP^+^ uptake was measured as described under Section 4. The graphs show representative experiments, respectively. K_i_ values shown in the context and Table 1 represent ± SEM (*n* ≥ 3). (**A**) Displacement of APP^+^ in TuriSERT WT by 5-HT and its precursors. (**B**) Displacement of APP^+^ by 5-HT in TuriSERT WT and its substrate binding site mutants.

The key residue Asp98 in hSERT, which has been demonstrated to interact with the primary amine of 5-HT and also to be completely conserved among biogenic amine transporters, is replaced by Gly22 in TuriSERT [37]. On the other hand, a glycine residue is highly conserved at this equivalent position among amino acid transporters in the NSS family. This raises the question of whether the amino acid precursors in 5-HT biosynthesis, such as tryptophan and 5-hydroxytryptophan, are good substrates for TuriSERT. To test this possibility, we examined the displacement of APP^+^ by 5-HT or its precursors in transport assay. Our data indicated that neither tryptophan nor 5-hydroxytryptophan showed a significant influence on APP^+^ uptake up to the highest concentration of 1 mM tested. In contrast, 5-HT effectively displaced APP^+^ with a K_i_ value of 23.93 ± 1.89 μM (Figure 4A), suggesting that 5-HT but not its amino acid precursors specifically bind to TuriSERT.

Comparison of the residues in hSERT orthosteric binding site with those in TuriSERT revealed several different residues, such as Tyr95 (Ser19 in TuriSERT), Asp98 (Gly22), Ala173 (Cys97), and Thr438 (Ala336), which have been indicated to interact with the tryptamine, primary amine, and hydroxyl groups of 5-HT molecules, respectively, in the cryo-EM structures of hSERT [18]. Substitutions of these residues in hSERT led to severe damage to its ability to transport 5-HT [19,40,41,42,43]. We, then, examined the effects of these residues in TuriSERT on 5-HT displacement of APP^+^ by switching them, one at a time, to the equivalent residues in hSERT. As shown in Table 1, all mutants exhibited comparable abilities to transport APP^+^ and similar K_i_ values for the 5-HT displacement of APP^+^ to those in WT, suggesting that these residues play distinctive roles in 5-HT binding between hSERT and its bacterial orthologue.

**Figure 5:**
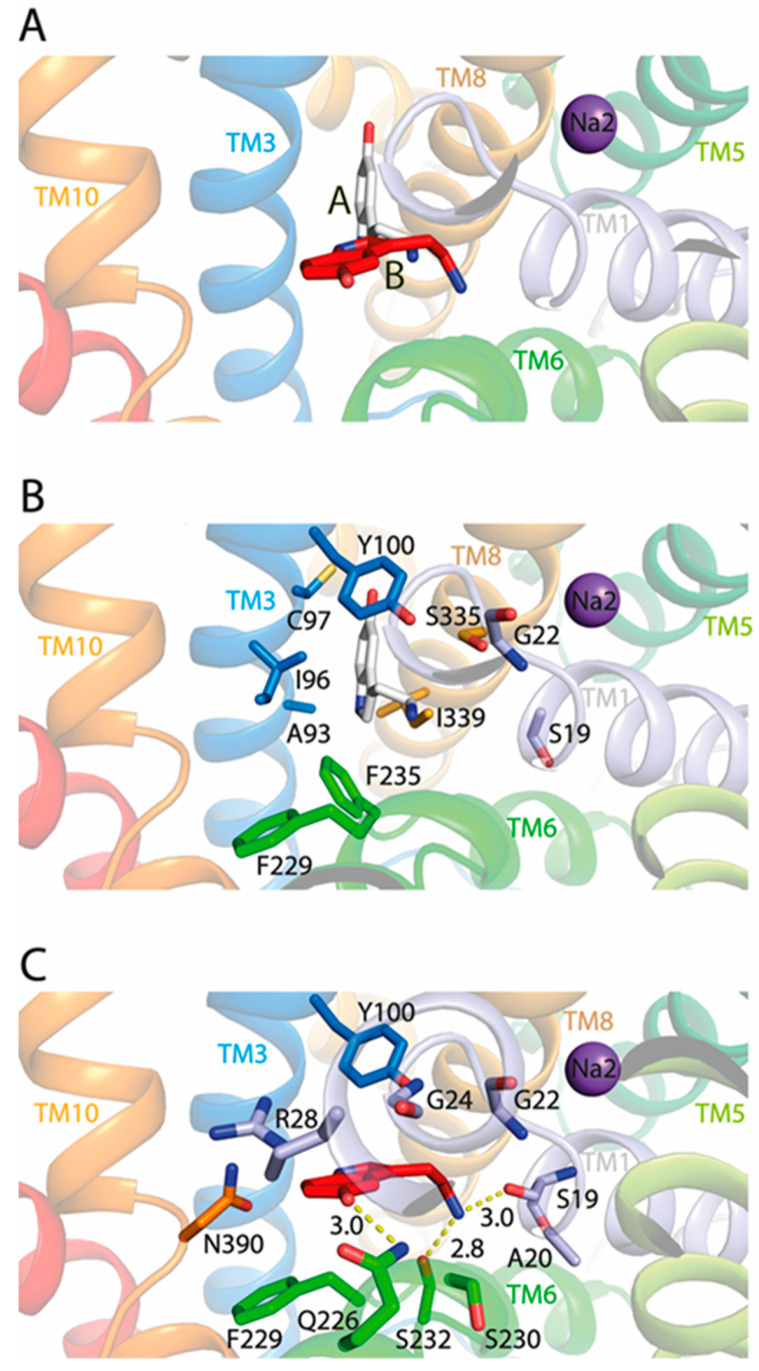
Close-ups of 5-HT binding in the TuriSERT-5-HT models. Silver and red molecules represent 5-HT in the proposed A and B sites, respectively. Purple spheres represent Na^+^ in the Na2 site. Residues that are proposed to contribute to 5-HT binding in TuriSERT are shown. (**A**) Two 5-HT binding poses in the TuriSERT models. (**B**) 5-HT binding in the proposed A site. (**C**) 5-HT binding in the proposed B site.

Next, we conducted the molecular docking of 5-HT to the TuriSERT model without Na^+^ binding in the corresponding Na1 site. Our docking generated two models of TuriSERT-5-HT complexes, in which the 5-HT molecule adopts two different binding poses in the bacterial transporter (Figure 5A). In one model, 5-HT exhibits a binding pose similar to the one found previously in the orthosteric binding site in the cryo-EM structures of the hSERT-5-HT complex (Figure 5B, named as the A site), whereas 5-HT shifts away from the orthosteric site with a protrusion into the corresponding Na1 site in another model (Figure 5C, named as the B site). Further inspection of the TuriSERT residues forming the 5-HT B site revealed that the tryptamine group of 5-HT is positioned between the aromatic groups of Tyr100 and Phe229, and the primary amine interacts with Ser19 and Ser232 through hydrogen bonds. The hydroxyl group of 5-HT inserts into the space between TM3 and TM6 and forms a hydrogen bond with the amide group of Gln226, a residue highly conserved in the NSS family [10].

To further validate the 5-HT binding site in TuriSERT, we selected several representative residues unique for 5-HT binding in the proposed A site (Ile96 and Phe235, Figure 5B), B site (Gln226 and Ser232, Figure 5C), or both (Tyr100 and Ser19, Figure 5B,C) for mutagenesis and monitored their influences on displacement potency of 5-HT. All mutants possessed comparable APP^+^ transport activity with slightly increased K_m_ values for APP^+^ relative to WT (Table 1). Notably, similar substitutions in hSERT, such as I172W (I96W in TuriSERT), Y176A (Y100A), and F341H (F235H) led to a loss of the ability to transport 5-HT [44]. Mutations of the residues that are supposed to interact with 5-HT molecules in both A and B sites, such as Y100A and S19V, led to a dramatic reduction in 5-HT displacement. 5-HT displacement potency was decreased by approximately five-fold in S19V, whereas little 5-HT displacement was detected in Y100A (Table 1 and Figure 4B). Strikingly, mutants in the A site, such as I96W and F235H, showed a displacement potency of 5-HT with less than a two-fold deviation from WT. In contrast, mutants in the B site, such as Q226A and S232A, exhibited little ability to displace APP^+^ by 5-HT in a transport assay (Table 1 and Figure 4B). These results suggest that 5-HT preferably binds to the B rather than the A site in TuriSERT.

### 2.6. Effect of Antidepressant Drugs on APP^+^ Transport

**Figure 6:**
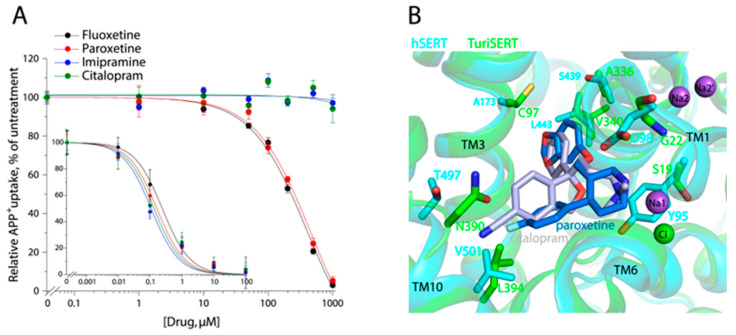
Antidepressant inhibition of APP^+^ uptake and drug binding poses. (**A**) Antidepressant inhibition of APP^+^ uptake by hSERT (insert) or TuriSERT. HeLa or *E. coli* cells expressing hSERT or TuriSERT were incubated with fluoxetine, paroxetine, citalopram, or imipramine at the indicated concentrations in the presence of APP^+^ for 5 min, and then APP^+^ uptake was measured as described under Section 4. The graphs show representative experiments with six measurements at each drug concentration. Error bars represent ± SD. The K_i_ values in the context represent ± SEM (*n* = 3). (**B**) Superimposition of the crystal structures of hSERT-citalopram (PDB, ID 5i71, 3.15 Å), hSERT-paroxetine (5i6x, 3.14 Å), and TuriSERT models. Silver and blue molecules represent citalopram and paroxetine, respectively. Purple and green spheres represent Na^+^ (Na1, Na2 in hSERT, and Na2′ in TuriSERT) and Cl^−^ in hSERT. Only the different residues at the equivalent positions between hSERT (cyan) and TuriSERT (green) are shown with the same colors as the corresponding transporters.

We examined the inhibition of APP^+^ transport activity of both hSERT and TuriSERT by several antidepressants, including SSRI antidepressants fluoxetine, paroxetine, and citalopram, and one tricyclic antidepressant imipramine. As shown in Figure 6A, all tested antidepressants effectively inhibited APP^+^ transport by hSERT with K_i_ values of 0.24 ± 0.05 μM for fluoxetine, 0.13 ± 0.02 μM for paroxetine, 0.26 ± 0.05 μM for citalopram, and 0.15 ± 0.05 μM for imipramine, respectively (Figure 6A, insert), although their inhibition potencies were much smaller than those in the inhibition of 5-HT transport. In contrast, these antidepressants showed distinctive effects on APP^+^ transport by TuriSERT. Fluoxetine and paroxetine inhibited transport activity with K_i_ values of 187.4 ± 5.1 μM for fluoxetine and 240.9 ± 11.0 μM for paroxetine, which are 780- and 1800-fold higher than those in inhibition of APP^+^ transport by hSERT, respectively, whereas two other antidepressant drugs, citalopram and imipramine, showed little inhibitory effect on APP^+^ transport by TuriSERT (Figure 6A).

To compare antidepressant binding between hSERT and TuriSERT, we superimposed the crystal structure of hSERT bound with paroxetine or citalopram with the TuriSERT model (Figure 6B). Several residues, such as Tyr95, Asp98, Ala173, Ser439, and Thr497, play critical roles in antidepressant recognition in hSERT [16,45]. Substitutions of these residues have been indicated to lead to a dramatic reduction in the inhibition potency of antidepressant drugs [43,44]. However, these key residues are replaced by Ser19, Gly22, Cys97, Ala336, and Asn390 in TuriSERT, respectively, perhaps explaining, in part, why antidepressants have only a slight inhibition against TuriSERT in comparison to hSERT.

## 3. Discussion

The present study characterized a bacterial orthologue of eukaryotic SERT using structural, biochemical, and pharmacological approaches. Our experimental results show that (i) Cl^−^ is not required for substrate transport by TuriSERT, (ii) substitutions of the corresponding Na1 site residues in TuriSERT do not alter Na^+^ binding dependence of substrate transport, and (iii) substitutions of several residues essential for substrate binding in eukaryotic amine transporters show little effect on 5-HT displacement potency in TuriSERT. These biochemical pieces of evidence support the proposal that the bacterial transporter, TuriSERT, transports its substrate with a novel substrate and ion recognition in comparison with other members of the NSS family.

The unique structural feature accounts for its distinctive substrate and ion recognition in TuriSERT. All resolved high-resolution structures of the NSS transporters have revealed the common binding sites for substrate and ions. The most striking structural characteristics of substrate and ion binding in these NSS family transporters are (i) the presence of an aspartate residue contributing to substrate binding in amine transporters and glycine at the corresponding position in amino acid transporters, (ii) the presence of a negatively charged residue (Glu290 in leucine transporter LeuT, Asp268 in tryptophan transporter TnaT, or Asp259 in tyrosine transporter Tyt1) in the corresponding Cl^−^ binding site in most prokaryotic NSS transporters, and (iii) the presence of two Na^+^ binding sites. These structural elements, together with other highly conserved residues in the binding sites, convey the ability of the NSS transporters to control conformational changes essential for Na^+^-coupled transport by facilitating the binding site interactions. In comparison with other NSS members, TuriSERT possesses a unique structural feature specific for amino acid binding but does transport an amine substrate [37]. Our displacement experiments also indicated that 5-HT but not its amino acid derivatives effectively displace the fluorescent substrate in APP^+^ transport assay for TuriSERT. Therefore, we propose that a unique structural feature elicits TuriSERT to adopt a novel configuration for coordinating its substrate and ions.

In the Cl^−^-independent prokaryotic NSS transporters, a negatively charged residue (Glu290 in LeuT or Asp269 in TnaT) is present at the position that corresponds to the Cl^−^ binding site in Cl^−^-dependent transporters. Replacing Glu290 in LeuT or the corresponding Asp269 in TnaT with serine renders a Cl^−^ requirement for substrate binding [38] or transport [39], whereas the reciprocal mutations in Cl^−^-dependent SERT, dopamine transporter (DAT), or γ-aminobutyric acid transporter 1 (GAT-1) to either glutamate or aspartate allow substrate transport in the absence of Cl^−^ [31,39]. Among the Cl^−^-independent transporters, Tyt1 and TuriSERT are unique because they have an aspartate (Asp259 in Tyt1 or Asp262 in TuriSERT) to replace an asparagine in the Cl^−^-dependent transporters at the position that corresponds to both the Na1 and Cl^−^ binding sites [37,46]. A mutation of Asp259 with asparagine in Tyt1 was previously shown to result in a Cl^−^-dependent transport phenotype [47]. Thus, a negatively charged residue in the Cl^−^ binding site has been proposed to prevent Cl^−^ from binding by constituting an electrostatic barrier in the Cl^−^-independent transporters [47]. A similar observation was also seen with a D262N mutant of TuriSERT in the present study, validating that the presence of a negative charge in the site, either a Cl^−^ ion or a carboxylate side chain is a unifying feature in all NSS transporters.

A negatively charged residue Asp262 in TuriSERT that renders the Cl^−^-dependent transport phenotype was also proposed to stabilize Na^+^ binding in the proximate Na1 site. However, the mutant D262N showed a comparable Na^+^ binding dependence of transport activity to that in WT. In addition, similar Na1 site substitutions (N25S or S230A in TuriSERT; N27S or T254A in LeuT) resulted in a significant difference in Na^+^ binding dependence of substrate binding or transport between LeuT and TuriSERT ([27], this study). In contrast, a similar Na2 site mutation (S335A in TuriSERT; S355A in LeuT) showed a consistent effect on Na^+^-dependent transport activity of both TuriSERT and LeuT ([27], this study). These results suggest that Na^+^ may bind only to the Na2 but not the Na1 site in TuriSERT. The absence of Na^+^ binding to the corresponding Na1 site is supported by our docking of a 5-HT molecule to the TuriSERT model in which 5-HT adopts a novel binding pose in the bacterial orthologue. Our docking showed that 5-HT shifts away from the S1 site with a protrusion into the corresponding Na1 site, possibly providing a steric barrier to Na^+^ binding in that site.

Our biochemical analysis supports the proposal that TuriSERT adopts a unique configuration to coordinate 5-HT. According to our mutagenesis, switching back to the corresponding residues essential for 5-HT binding in hSERT, especially Ser19 to phenylalanine, Gly22 to aspartate, or Ala336 to threonine, did not significantly change the 5-HT displacement potency in the APP^+^ transport assay for TuriSERT. Additionally, similar mutations of two other critical residues for 5-HT binding in the S1 site (Ile172 with tryptophan or Phe341 with histidine in hSERT; Ile96 with tryptophan or Phe235 with histidine in TuriSERT) led to inactive hSERT, which was possibly due to a poor 5-HT binding ability [44], but a negligible effect on the ability of 5-HT to displace APP^+^. These results suggest that 5-HT binds to a novel site rather than the S1 site in TuriSERT. To further identify the 5-HT binding site in TuriSERT, we mutated several residues that are indicated to coordinate 5-HT in our docking model. 5-HT displacement potency was dramatically reduced in all four mutants tested, of which three mutants (Y100A, Q226A, and S232A) lost the ability to bind with 5-HT, whereas another (S19V) showed an approximate five-fold decrease of 5-HT displacement potency relative to that in WT, which is consistent with the proposed coordination for 5-HT binding in TuriSERT in our docking model. Asp262 in TuriSERT has been speculated to play a similar role to Asp98 in hSERT by interacting with the primary amine group of 5-HT [37]. However, compared to other mutations in the proposed 5-HT binding site, the reciprocal substitution of Asp262 with asparagine only resulted in a slightly decreased ability of 5-HT to displace APP^+^, arguing that this acidic residue may not be capable of directly participating in 5-HT binding.

The present study has demonstrated the existence of a unique substrate binding site and 1:1 stoichiometry of sodium–substrate binding in TuriSERT, which is a novel structural finding for the transporters in the NSS family. For Cl^−^-independent NSS transporters, H^+^ antiport has been proposed to be involved in substrate transport for maintaining charge balance in Tyt1 and MhsT [21,47]. Mutants of Tyt1 and MhsT in which an aspartate in the corresponding Cl^−^ site was substituted by asparagine (D259N in Tyt1 and D263N in MhsT) are Cl^−^-dependent and show diminished H^+^ antiport [47]. TuriSERT also has an aspartate at the corresponding position (Asp262), suggesting that Na^+^-coupled substrate symport by TuriSERT can be stimulated by a similar mechanistic feature.

In contrast to other members in the NSS family with a 2:1 stoichiometry of sodium–substrate binding, TuriSERT binds only one Na^+^ ion at the Na2 site. An early study of mammalian SERT in platelet plasma membrane vesicles has demonstrated 5-HT transport with only one Na^+^ ion [48]. In addition, structural analysis of mammalian biogenic amine transporters in various conformational states has proposed that only the Na^+^ at Na2 dissociates to the cytoplasm when the transporters transition to the inward-open conformation, whereas the Na^+^ ion at Na1 remains bound to the transporters during the transport process, leading to a 1:1 stoichiometry of sodium–substrate transport [28]. Hence, it is reasonable to assume that TuriSERT shares a 1:1 stoichiometry of sodium–substrate transport with mammalian biogenic amine transporters, although they bind different numbers of Na^+^ ions. However, the lack of the Na^+^ at Na1 in TuriSERT implies that it must take a different molecular solution to satisfy requirements for ensuring the binding site interactions and subsequently for controlling conformational changes essential for Na^+^-coupled substrate transport. Hence, future elucidation of the mechanistic features of TuriSERT is expected to improve our understanding of the transport mechanism of the NSS family transporters.

Our results indicated that all tested antidepressants have little or a slightly inhibitory effect on APP^+^ transport by TuriSERT, which is apparently due to the substitutions of several residues essential for coordinating these drugs in hSERT. The small difference in the inhibitory potency (K_i_) against TuriSERT between these drugs is probably due to the variation in the interactions between these antidepressant molecules and their binding sites in TuriSERT. Because TuriSERT participates in the regulation of the 5-HT level in the gut, which plays dual roles (conventional and nonconventional) in the physiology of the gut [37], agents antagonizing or stimulating its transport activity may be medically beneficial.

## 4. Materials and Methods

### 4.1. Expression Plasmids

The TuriSERT gene was synthesized according to a full-length nucleotide sequence encoding SERT of *T. sanguinis* reported in the GenBank (accession number ZP_06621923) and then cloned into an *E. coli* expression vector pGEX under the control of the tac promoter using the ClonExpress II One Step Cloning Kit (Vazyme, Nanjing, China). The expression plasmid pGEX-TuriSERT contains sequences encoding a GST tag at the *N*-terminus and a 6 x His epitope tag at the C-terminus of TuriSERT. Mutant transporters were generated with pGEX-TuriSERT as a template by site-directed mutagenesis using the Mut Express II Fast Mutagenesis Kit V2 (Vazyme, Nanjing, China). All mutations were confirmed by full-length DNA sequencing.

### 4.2. Expression of TuriSERT

*E. coli* C43(DE3)/pLysS cells (Thermo Fisher, Waltham, MA, USA) transformed with the expression plasmid pGEX-TuriSERT were grown in Luria–Bertani (LB) broth containing 100 μg/mL ampicillin and 34 μg/mL chloramphenicol at 37 °C with shaking at 200 rpm. Expression of TuriSERT was induced by adding 1 mM isopropyl-β-D-thiogalactoside (IPTG) to the cell culture at an OD_600_ of 0.8 and subsequently incubating at 16 °C for an additional 18 h. Protein concentration was determined with the Micro BCA protein assay reagent kit (Thermo Fisher, Waltham, MA, USA).

### 4.3. Homology Modeling and Molecular Docking

A homology model of TuriSERT was generated with Modeller under Pymod3 version 3.0.2 (https://schubert.bio.uniroma1.it/pymod/index.html) [49] based on an outward structure (3.3 Å resolution, PDB ID 7LIA) of hSERT. The alignment of two amino acid sequences was obtained with ClustalX2 version 2.1 (http://www.clustal.org/clustal2/). The sequence identity between TuriSERT and hSERT is 34.15%, which is expected to result in an accuracy of 0.7–2.0 Å for the backbone of the transmembrane regions of this model [37,50]. In addition, residues contributing to 5-HT and ion binding sites in the core region of hSERT are ~50% identical and ~70% similar to those in TuriSERT, and the residues required to bind a Na^+^ ion at the Na2 site are identical (Supplementary Figure S1). Therefore, the homology model of TuriSERT based on its mammalian orthologue, hSERT, is highly expected to provide useful structural information for characterizing the bacterial transporter. The model with the highest score (DOPE score, −76,207) was selected for visualization. Another model of TuriSERT was also generated by using AlphaFold2 version 1.5.3 (Google DeepMind, Mountain View, CA, USA) [51]. Two models of TuriSERT superimposed almost seamlessly, except for two nonessential regions for the catalytic function, TM12 and IL5 (Supplementary Figure S2); thus, we performed molecular docking using the homology model of TuriSERT based on the structure of hSERT. Figures of the TuriSERT model were generated using PyMOL version 2.5.2 (Schrödinger Inc., New York, NY, USA).

Molecular docking was carried out with AutoDock Vina 1.1.2 on the structure of the TuriSERT model with Na^+^ binding only in the Na2 site. The template structure and substrate were then subjected to automated structure preparation using AutoDockTools in order to optimize the hydrogen bonding network, conformation of bonds, and energy constraints. A 5-HT molecule was input into AutoDock Vina and optimized for its conformation and energy in the Vina force field [52]. Docking was performed using AutoDock Vina under a standard precision (exhaustiveness) with the ligand in a flexible conformation. In the docking step, 20 poses for 5-HT in the TuriSERT model were generated. 5-HT posing with the residues within 5 Å was subject to conformational search and energy minimization. The refined TuriSERT-5-HT complexes were ranked by affinity scores. The more negative the affinity score was, the more favorable 5-HT binding to the residues was. The top two poses with the lowest energy for the TuriSERT-5-HT complex were exported into PyMOL for visualization.

### 4.4. APP^+^ Uptake Assay

APP^+^ uptake by TuriSERT was measured using *E. coli* cells transformed with pGEX-TuriSERT. In brief, the cells grown in 10 mL LB medium were washed three times with 600 μL of KRH buffer containing 20 mM HEPES, pH 7.4, 120 mM NaCl, 1.3 mM KCl, 2.2 mM CaCl_2_, 1.2 mM MgSO_4_, and 0.1% (*w*/*v*) glucose. APP^+^ uptake was measured by adding 600 μL of KRH buffer containing 6 μM APP^+^ and incubating for 5 min at 37 °C. The assays were terminated by three rapid washes with 600 μL of ice-cold KRH buffer. The cells in 600 μL of KHR buffer were transferred into a 96-well plate, with 100 μL per well. The extent of APP^+^ accumulated in the cells was determined with the Infinite 200 Pro Microplate Reader (Tecan, Grödig, Austria). The excitation wavelength for APP^+^ was 488 nm, while the emission filter was set at 525 nm for APP^+^. Nonspecific transport was measured in the presence of 1 mM fluoxetine. To measure the ionic effects on the APP^+^ uptake, 150 mM *N*-methyl-D-glutamine (NMDG) gluconate and NMDG chloride, sodium isethionate, or NaCl were added to 20 mM HEPES buffer, pH 7.4, as indicated.

APP^+^ transport by hSERT was measured as described previously [53,54]. In brief, HeLa cells stably expressing hSERT were wet mounted on polylysine-coated glass slides and applied for the indicated treatments. The cells were washed twice with 500 μL KRH buffer and incubated with 2 μM APP^+^ in KRH buffer for 5 min at 22 °C. The reactions were stopped by 3× rapid washes with 500 μL ice-cold KRH buffer. The extent of APP^+^ accumulated in the cells was determined with confocal imaging analysis. Nonspecific transport for APP^+^ was measured in the presence of 10 μM fluoxetine and subtracted to give the APP^+^ transport activity.

### 4.5. Data Analysis

Nonlinear regression fits of experimental and calculated data were performed with Origin 2021 version 9.8.0.200 (Origin Lab, Northampton, MA, USA). The statistical analysis given is based on multiple experiments. Data with error bars in the figures represent the mean ± SD for 6 measurements per condition in one experiment or the mean ± SEM for three experiments as indicated, respectively. Statistical analysis was performed using Student’s paired *t* tests.

## 5. Conclusions

The present study characterized a bacterial orthologue of mammalian SERT, TuriSERT, which possesses a striking structural feature, by using biochemical, structural, and pharmacological approaches. Our results indicated that TuriSERT adopts a unique configuration to coordinate the substrate 5-HT, with a 1:1 stoichiometry of sodium–substrate binding, which is a novel structural finding for the transporters in the NSS family. In addition, our results also demonstrated that SSRI antidepressants exert a weak inhibitory effect on TuriSERT. The present study provides new insights into substrate/ion binding and transport mechanisms of the important NSS transporter family.

## Figures and Tables

**Table 1:** Kinetic analysis for TuriSERT and its mutants and displacement of APP^+^ by 5-HT.

Binding Site		K_m_ for APP^+^ (μM)	V_max_ (AFU/mg/min)	K_m_ for Na^+^ (μM)	K_i_ for 5-HT (μM)
	WT	6.12 ± 0.27	1285 ± 14	33.33 ± 1.32	26.13 ± 2.21
Na1 or Cl^−^	N25S	12.78 ± 1.22 *	857.4 ± 46.08 *	42.41 ± 0.92 *	36.25 ± 0.72
	S230A	13.66 ± 0.60 *	902.5 ± 17.81 *	37.49 ± 0.81	37.82 ± 1.47
	D262N	14.61 ± 1.43 *	841.4 ± 22.3 *	21.56 ± 0.82 *	65.03 ± 7.53 *
Na2	S335A	NF	NF	NF	NF
5-HT	S19Y	10.47 ± 0.40 *	1205 ± 53.42	ND	30.72 ± 1.78
	G22D	10.29 ± 0.54 *	593.2 ± 59.4 *	32.33 ± 0.94	15.63 ± 0.83
	C97A	9.93 ± 1.16	1195 ± 7.75	ND	30.77 ± 1.63
	A336T	9.23 ± 1.12	1431 ± 145.1	ND	37.43 ± 1.42
	S19V	18.22 ± 0.44 **	1127 ± 23.99	ND	124.32 ± 3.31 **
	Y100A	19.17 ± 1.99 **	773.2 ± 89.66 *	ND	>1000
	I96W	9.12 ± 1.52	1294 ± 175.9	ND	41.21 ± 2.16
	F235H	12.71 ± 0.35 *	1051 ± 9.82	ND	33.77 ± 1.89
	Q226A	22.32 ± 0.51 **	1292 ± 51.22	ND	>1000
	S232A	16.24 ± 0.64 **	1163 ± 34.43	ND	>1000

Kinetic analysis for APP^+^ or Na^+^ was performed with *E. coli* cells expressing TuriSERT or its mutants in KRH buffer containing APP^+^ at a range of concentrations (0.1–20 μM) or in 20 mM HEPES buffer, pH 7.4, containing 6 μM APP^+^ and NaCl at various concentrations (1–200 mM) supplemented by NMDGCl to keep the same ionic molarity in each reaction, respectively. 5-HT displacement of APP^+^ was measured by incubating *E. coli* cells with APP^+^ and 5-HT at a range of concentrations (1–100 μM). K_m_ values for APP^+^ and Na^+^, V_max_ values, and K_i_ values for 5-HT displacement represent ± SEM (*n* ≥ 3). * *p* < 0.05; ** *p* < 0.01 compared to WT. NF, nonfunctional; ND, not determined.

## Data Availability

The data presented in this study are available on request from the corresponding author.

## Supplementary Materials

The following supporting information can be downloaded at: https://www.mdpi.com/article/10.3390/ijms242317112/s1.
