# Supplementary material for: Unique Substrate Recognition and Sodium–Substrate Binding Stoichiometry in a Bacterial Serotonin Transporter, TuriSERT
Source: Int J Mol Sci. 2023 Dec 4;24(23):17112. doi: 10.3390/ijms242317112 (PMC10707687; doi:10.3390/ijms242317112)
Supplement: Supplementary file 1 [file ijms-24-17112-s001.zip › ijms-2722661-supplementary.pdf]

Supplementary Figures

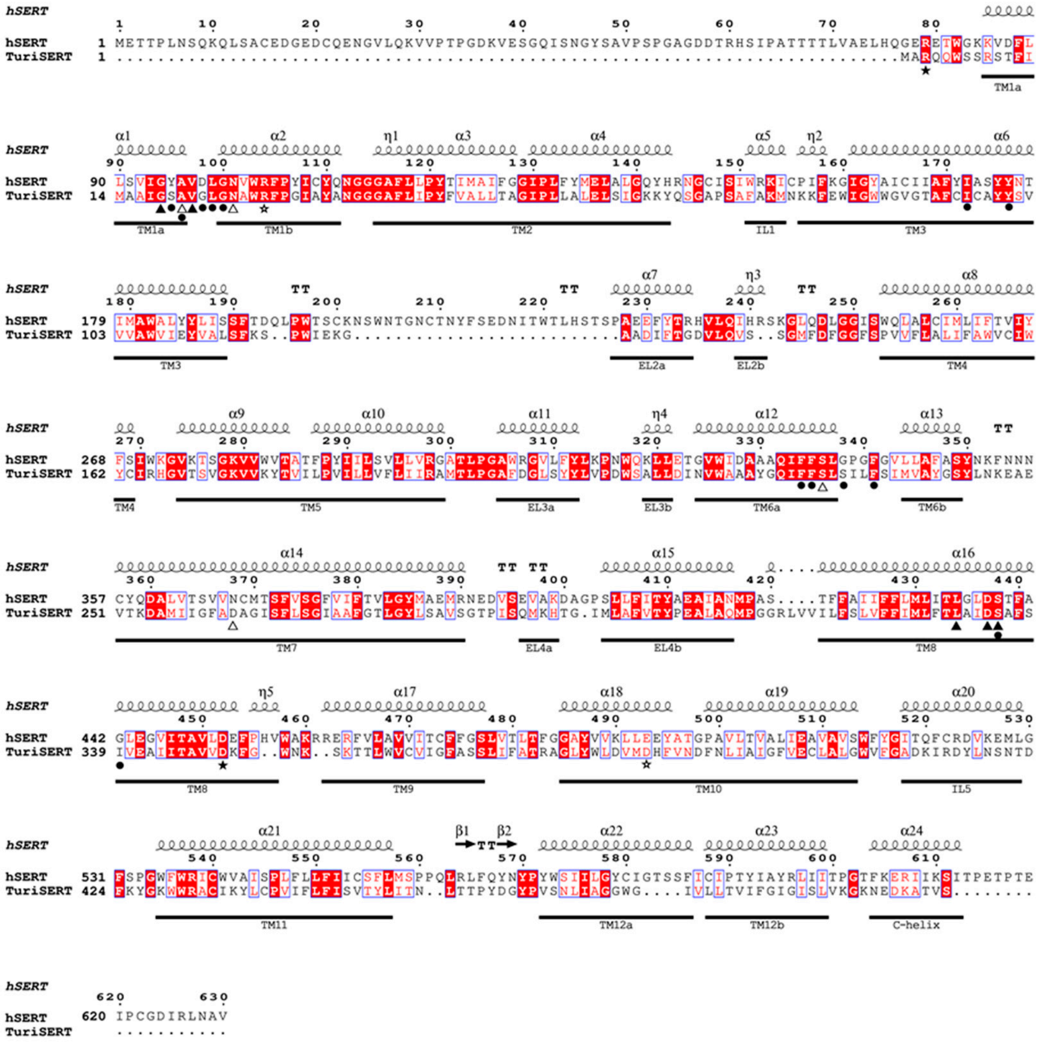

Figure S1. Amino acid sequence alignment of hSERT with TuriSERT. The sequence alignment was obtained with ClustalX2. Identical residues are highlighted in white letters with a red background and similar residues are shown in red letters. α-Helices and β-strands in hSERT are depicted as coils and arrows respectively. The open and filled triangles show residues coordinating sodium ions Na1 and Na2, respectively. The filled black circles indicate the residues involved in 5-HT binding in hSERT. The open and filled stars indicate the ion pairs at the extracellular and cytoplasmic entrances, respectively.

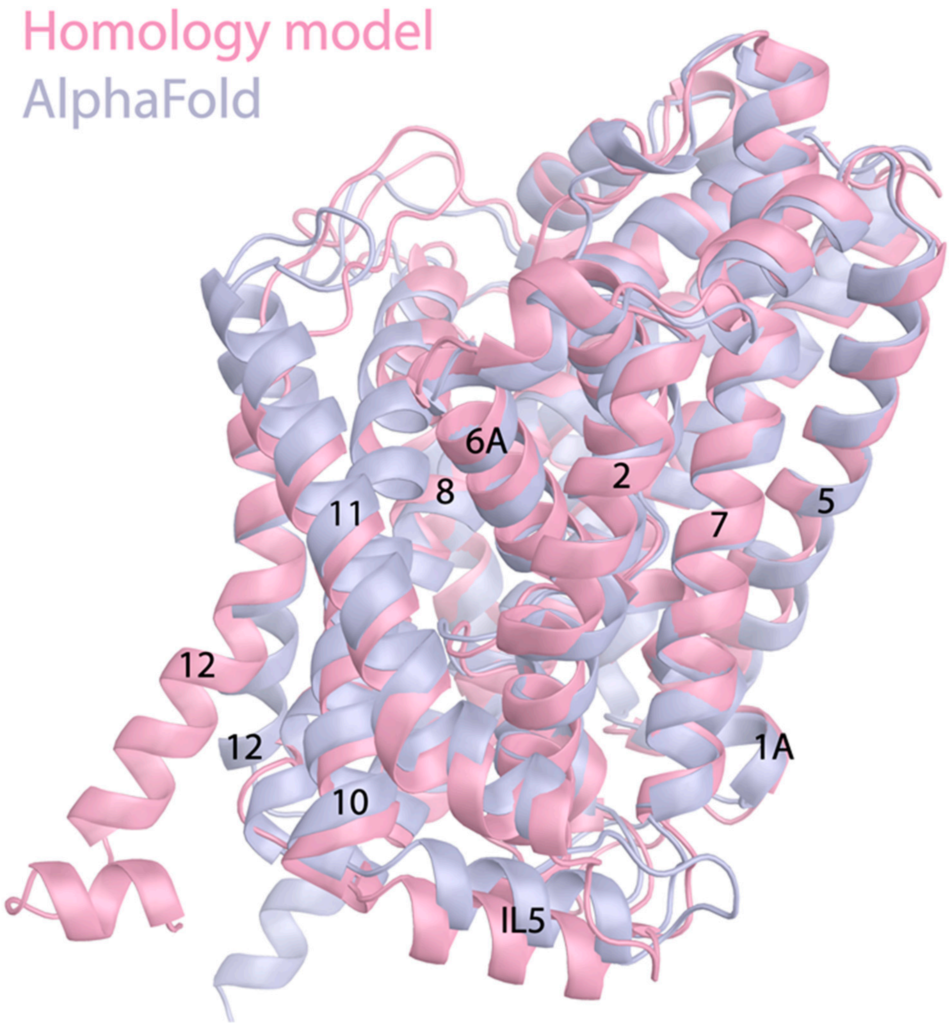

Figure S2. Superimposition of TuriSERT models generated by homology modeling (light pink) and AlphaFold2 (blue white). Numbers represent # of transmembrane α-helice in transporters.
